# Healthcare costs related to respiratory syncytial virus in paediatric intensive care units in the Netherlands: a nationwide prospective observational study (the BRICK study)

**DOI:** 10.1016/j.lanepe.2024.100965

**Published:** 2024-06-26

**Authors:** Emily W.E.M. Phijffer, Joanne G. Wildenbeest, Carole N.M. Brouwer, Matthijs de Hoog, Martin C.J. Kneyber, Sofie Maebe, Anneliese Nusmeier, Maaike A. Riedijk, Roelie M. Wösten-van Asperen, Job B.M. van Woensel, Louis J. Bont, Geert J.W. Frederix

**Affiliations:** a Department of Paediatric Infectious Diseases and Immunology, Wilhelmina Children's Hospital, University Medical Center Utrecht, the Netherlands; b Division of Paediatric Intensive Care, Department of Neonatal & Paediatric Intensive Care, Erasmus MC – Sophia Children's Hospital, Rotterdam, the Netherlands; c Paediatric Intensive Care Unit, Maastricht University Medical Centre, Maastricht, the Netherlands; d Division of Paediatric Critical Care Medicine, Department of Paediatrics, Beatrix Children's Hospital, University Medical Centre Groningen, Groningen, the Netherlands; e Paediatric Intensive Care Unit, Leiden University Medical Centre, Leiden, the Netherlands; f Paediatric Intensive Care Unit, Amalia Children's Hospital, Radboud University Medical Centre, Nijmegen, the Netherlands; g Paediatric Intensive Care Unit, Wilhelmina Children's Hospital, University Medical Center Utrecht, the Netherlands; h Paediatric Intensive Care Unit, Emma Children's Hospital, Amsterdam UMC Location University of Amsterdam, Amsterdam, the Netherlands; i Julius Center for Health Sciences and Primary Care, University Medical Center Utrecht, Utrecht, Netherlands

**Keywords:** Respiratory syncytial viruses, Critical care, Tertiary care centers, Cost of illness, Healthcare costs

## Abstract

**Background:**

The implementation of the approved respiratory syncytial virus (RSV) preventive interventions in immunisation programmes is advancing rapidly. Insight into healthcare costs of RSV-related paediatric intensive care unit (PICU) admissions is lacking, but of great importance to evaluate the impact of implementation. Therefore, this study aimed to determine the total annual RSV-related paediatric intensive care healthcare costs in the Netherlands.

**Methods:**

A nationwide prospective, observational, multicenter study was performed from September 2021 until June 2023. The total annual RSV-related healthcare costs on PICUs in the Netherlands were calculated using RSV-related costs (subgroup I) and consequential costs (subgroup II and III). Subgroup I comprised all PICU admitted infants ≤12 months of age with laboratory-confirmed RSV infection. Subgroup II and III consisted of postponed elective PICU admissions and refused acute PICU admissions due to RSV-related lack of PICU capacity.

**Findings:**

A total of 424 infants with RSV-related PICU admission were included. Median age at PICU admission was 46 days (IQR 25–89). The median length of PICU admission was 5 days (IQR 3–8). The total RSV-related PICU costs are € 3,826,386 in 2021–2022, and € 3,183,888 in 2022–2023. Potential costs averted by RSV preventive interventions is € 1.9 to € 2.6 million depending on season, and the duration of protection.

**Interpretation:**

RSV-related PICU admissions cost €3.1 to €3.8 million in the Netherlands during one season. The introduction of new RSV preventive interventions into the Dutch immunisation programme will generate significant cost-savings on PICUs and decreases the admission burden of PICUs.

**Funding:**

None.

**Research in context**

**Evidence before this study**

RSV preventive interventions, such as maternal vaccination or infant immunisation via monoclonal antibodies (mAbs) can have a profound impact on RSV-related PICU admissions. Currently, the EMA's Committee for Medicinal Products for Human Use and the FDA approved the extended half-life mAb (nirsevimab) and the maternal RSV prefusion F subunit vaccine. The implementation of the approved RSV preventive interventions in immunisation programs is advancing rapidly. Decisions regarding implementation are apart from health economic outcomes of individual interventions also informed by the potential impact and the healthcare costs averted. The Netherlands are expected to decide on implementation of a new RSV preventive intervention in the coming years.

On 9 June 2021, we searched PubMed using the terms “RSV OR respiratory syncytial virus” AND “hospitalisations OR hospitalizations OR intensive care” AND “costs” for studies between 1 January 1993 and 9 June 2021 with no language restrictions. We found 173 studies, of which only some performed a cost of illness study in other high income settings. One study performed a cost of illness study including intensive care admissions in Australia. In the Netherlands a cost-effectiveness analysis was performed in 2012 assessing societal costs of RSV. However, this analysis is primarily based on data about hospital admissions, since limited data is available about RSV-related PICU admissions. Therefore, potential health-economic impact of PICU admissions are not yet fully understood.

**Added value of this study**

This is the first nationwide, prospective, observational study to define healthcare costs of RSV-related PICU admissions in the Netherlands. We selected costs of RSV infection and consequential impact defined as postponed elective admissions and transfers. We found 236 RSV-related PICU admissions during the first season and 188 RSV-related PICU admissions during the second season. The total RSV-related healthcare costs on PICUs was estimated to be € 3.1 to 3.8 million in the Netherlands per RSV season. These healthcare costs were primarily related to infants admitted to the PICU with RSV. Assuming a vaccine efficacy of 80% and a vaccine uptake of 100% we show that the potential PICU related healthcare costs averted by RSV preventive interventions vary from €1.9 to €2.6 million per season depending on the RSV season and the duration of protection.

**Implications of all the available evidence**

This study provides the RSV-related healthcare costs on the PICUs in the Netherlands during two RSV seasons. The introduction of two newly approved RSV preventive interventions into the Dutch immunisation program will generate significant cost-savings on PICUs. Future, prospective research combining primary care cases, hospitalisations and PICU admissions is important to understand the full impact of RSV preventive interventions in the Netherlands.

## Introduction

Respiratory syncytial virus (RSV) is the most common cause of lower respiratory tract infections (LRTI) during infancy with a peak incidence of infants aged younger than six months.^1^ It has been estimated that RSV causes 33.3 million acute LRTI's in young children annually worldwide, with 3.6 million children requiring hospitalisation worldwide.^1^ If a paediatric intensive care unit (PICU) is available, approximately 5% of the hospitalised infants require PICU admission because of severe RSV disease.^2^^,^^3^ Every year the available PICU capacity is stretched to the limit during the RSV season.^4^

RSV preventive interventions, such as maternal vaccination or infant immunisation via monoclonal antibodies (mAbs) may have a profound impact on RSV-related PICU admissions.^5^ Currently, twenty RSV preventive interventions are under clinical development,^6^ and already two gained market approval. The EMA's Committee for Medicinal Products for Human Use and the FDA approved the extended half-life mAb (nirsevimab), and the maternal RSV prefusion F subunit vaccine.^7^, ^8^, ^9^, ^10^ Both, the mAb and the maternal vaccine showed safety and efficacy in infants. The maternal vaccine showed a vaccine efficacy of 67.7% (99.17% CI, 15.9–89.5) in preventing RSV-related hospitalisations within 90 days after birth. In addition, the mAb showed a vaccine efficacy of 77.3% (95% CI, 50.3–89.7) in preventing RSV-related hospitalisations in healthy preterm and term infants.^11^^,^^12^

The implementation of the approved RSV preventive interventions in immunisation programmes is advancing rapidly. Some European countries already offer nirsevimab to all infants below six months of age before the RSV season or infants born during the RSV season.^13^, ^14^, ^15^ It is expected that a new RSV preventive intervention will be implemented in the Netherlands in the coming years.^16^ However, if new preventive interventions for all infants will be implemented, more insight in the health-economic potential regarding savings and subsequent impact is important. Knowledge of the health-economic impact will allow decision makers to better define the maximum price at which society accepts buying future preventive interventions, the “willingness to pay”. The health-economic impact of both new interventions has been studied in European cost-effectiveness analyses recently.^17^^,^^18^ However, these analyses are based on data about RSV burden primary care and hospital admissions. Only limited data is available about RSV-related PICU admissions because this information is difficult to extract from available databases. Therefore, health-economic benefits on PICUs are not yet fully understood, while PICU admissions are associated with the highest costs. Therefore, insight into healthcare costs of RSV-related PICU admissions is of great importance to evaluate the impact of implementation of RSV preventive interventions, as well as consequential costs of RSV-related PICU admissions, and future PICU resource and capacity planning.

This study aims to determine the total annual RSV-related PICU healthcare costs in the Netherlands during two seasons of RSV surveillance.

## Methods

### Design

A nationwide, prospective, observational, multicenter study was performed from September 2021, until June 2023 in the Netherlands. All seven Dutch PICUs participated.

### Study population

The BRICK (Burden of RSV-related Intensive Care admissions of Kids) study was initiated in September 2021 aiming to collect data during two RSV seasons (2021–2022 and 2022–2023). Because of continuous RSV circulation, data collection for the first season was extended until September 2022 and was directly followed by the second season, starting in October 2022 and ending mid June 2023.

**Fig. 1:**
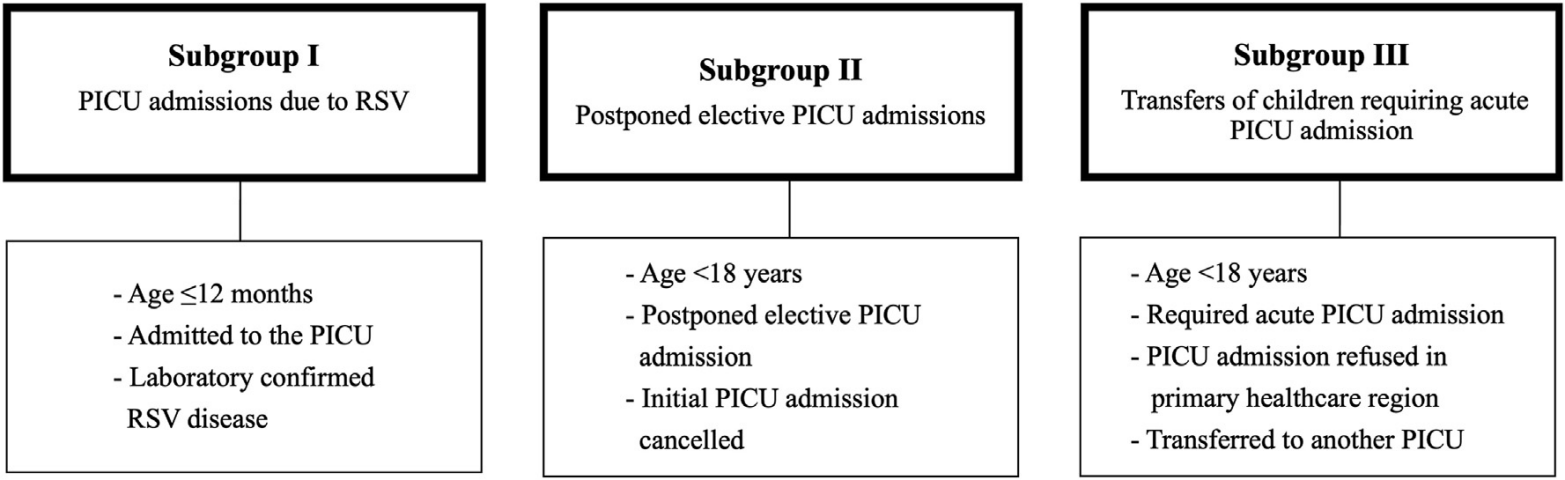
BRICK study subgroups. PICU, paediatric intensive care unit, RSV, respiratory syncytial virus. Patients in subgroup II and III are consequential to patients in subgroup I.

The study population was divided into three subgroups (Fig. 1). Subgroup I comprised all patients admitted to the PICU (≥ one day) with a laboratory-confirmed RSV infection ≤12 months of age. Subgroup II comprised all postponed elective PICU admissions and subgroup III comprised all acute PICU admissions requiring a transfer to another PICU location. We included patients in subgroup II and III in case at least one patient of subgroup I was admitted at the same PICU at the same time. We hypothesized that if at least one patient in subgroup I was admitted, and there was a lack of PICU capacity for other patients requiring PICU admission, this was attributable to RSV.

### Data collection

Study collaborators shared data weekly. The following data related to the PICU admission of subgroup I was obtained from electronic health records (EHRs): transport type before PICU admission (e.g. when transported from home or another hospital) including mobile intensive care unit (MICU) or ambulance, demographics, length of PICU stay, transport at PICU discharge, length of PICU readmission stay related to the initial admission up to one year after discharge, number of follow-up outpatient clinic visits with or without neuropsychological assessment (NPSA) up to one year after discharge. An outpatient clinic visit with NPSA is performed by a paediatric intensive care specialist and/or a psychologist to determine long term psychological sequelae in both parents and infants at 3–6 months after discharge. Duplicates resulting from readmissions for the same disease episode were removed.

For subgroup II the primary indication for elective admission (e.g. cardiac surgery) and the length in days between the primary elective admission and the newly scheduled elective admission were obtained. For subgroup III the type of transfer (MICU or regular ambulance) and origin and destination of transfer were obtained. All individual cases of subgroups II and III were crosschecked with cases in subgroup I to verify the patient was transferred or postponed at the time that at least one RSV patient of subgroup I was admitted to the PICU. In addition, we assessed whether the transfer details for patients in subgroup III mirrored those for patients in subgroup I, indicating that the data were accidentally collected for transport of a patient with RSV disease and therefore the same individual. Duplicates were removed. All data were entered into Castor EDC.^19^

### Total RSV-related healthcare costs

The primary endpoint of the study was the total annual RSV-related healthcare costs at PICUs in the Netherlands. The costs were calculated per infant from a hospital perspective and expressed in euros (€). Health economic guidance published by the Dutch Health Care Institute (ZIN) was used. The ZIN costing manual was used to obtain individual unit costs.^20^^,^^21^

We selected the most essential costs based on earlier evaluations related to RSV disease^22^ including costs of RSV infection and consequential costs defined as postponed elective admissions and transfers. In our cost calculation, we used unit costs. In case unit costs were unknown we used diagnosis–treatment combination (DBC) costs (Table S1). The Dutch Healthcare Authority (NZa) develops DBC products on an annual basis.^23^ Costs of DBCs represent up-to-date total costs per healthcare product. To determine the potential impact of new RSV preventive interventions in the current situation, we extrapolated and expressed all costs in Euros for the year 2023.

We defined costs of subgroup I as all costs that are directly attributable to RSV infection. First, costs of subgroup I included transport costs before admission. Transport costs were calculated as the type of transport before PICU admission multiplied by the unit costs of the transport type (MICU or regular ambulance). Second, costs of PICU admissions of patients in subgroup I were calculated as the total number of PICU admission days multiplied by the unit costs of one PICU admission day. Third, costs of ambulance transports at discharge were calculated as the ambulance transport at PICU discharge multiplied by the unit costs of the transport type (ambulance). Fourth, costs of PICU readmissions were calculated as the total number of PICU readmission days (after primary PICU admission) multiplied by the unit costs per PICU admission day. Fifth, costs of outpatient clinic visits were calculated as the total number of outpatient clinic visits (after primary PICU admission) with or without (NPSA) multiplied by the DBC costs of a paediatric post-IC outpatient clinic visit.

We defined costs of subgroup II and III as costs that are a consequence of patients in subgroup I. Subgroup II includes all medical costs for postponing the elective PICU admission because of insufficient capacity if at that moment at least one infant of subgroup I was admitted. E.g. increase of required medication or length of hospital admission because of increase in disease severity. No unit costs or DBC (diagnosis–treatment combination) is available for postponed elective PICU admissions. Therefore, we described the data. Subgroup III includes the total number of transfers of patients requiring acute PICU admission to another PICU due to insufficient capacity at their primary PICU if at that moment at least one RSV patient was admitted multiplied by the unit costs of the type of transfer (MICU or ambulance).

### Assumptions

If the type of transfer in subgroup I (at admission) and subgroup III was missing, we assumed the type of transfer was the least expensive (transport by regular ambulance).

If type of transfer after discharge in subgroup I was missing, we assumed that no transfer was required after PICU admission. For example, when the infant was discharged home after admission.

If NPSA during the outpatient clinic visit was not mentioned or missing, we assumed no NPSA was performed.

If data were missing, the median per patient costs were imputed. This was the case for one patient only.

### Impact analysis

The secondary endpoint of the study was to predict the potential maximum impact of future RSV preventive interventions (passive immunisation and maternal vaccination) using individual age at PICU admission, and the expected effect of both RSV preventive interventions on subgroup I. Therefore, we assumed both RSV preventive interventions have a consistent efficacy of 80% in reducing RSV-related hospitalisations in infants from birth up to three or six months of age^11^^,^^12^ and pregnant women and/or their infants have a 100% vaccine uptake. In addition, we performed sensitivity analysis on the impact of RSV preventive interventions using a hypothesized minimal effect with 60% vaccine efficacy in reducing RSV-related hospitalisations and 70% vaccine uptake.

### Ethical approval

This study is conducted in accordance with the principles and national guidelines of Good Clinical Practice in the Netherlands. The institutional research board of the University Medical Centre Utrecht waived the requirement for informed consent as only secondary anonymous data will be collected and analyzed.

### Statistical analyses

Characteristics of RSV-related PICU admissions were described using summary estimates, are expressed as median (interquartile range) and proportions (%). Where appropriate Chi-square tests and nonparametric tests (Mann Whitney-U) were used. Data were analyzed using Excel version 16.81 and SPSS (Version 29.0.1).

### Role of funding source

None.

## Results

### Baseline characteristics subgroup I

A total of 424 infants admitted to the PICU with confirmed RSV were included. Of one infant data about PICU admission, transport and follow-up were missing. Descriptive characteristics are shown in Table 1. The median age at PICU admission was 46 days (IQR 25–89). In total, 319 (75.2%) infants were below three months and 385 (91.0%) below six months of age. Before PICU admission 401 (94.8%) infants were transferred, of which 194 (45.8%) MICU transports, and 207 (48.8%) regular ambulance transports. The median length of PICU admission was 5 days (IQR 3–8). Eighteen (4.2%) infants required PICU readmission after discharge, median length of PICU readmission stay was 4 days (IQR 1–6). In total 168 (39.6%) infants were seen at the follow-up outpatient clinic visit, of which 120 (71.0%) also had a NPSA.

**Table 1:** Descriptive characteristics of infants admitted to PICU with RSV (subgroup I).

Characteristics	Season 1 (Sep 2021–Sep 2022)	Season 2 (Oct 2022–June 2023)
	PICU admitted infants (n = 236)^a^	PICU admitted infants (n = 188)
Age at PICU admission (median days, IQR)	45 (24–93)	49 (28–83)
≤6 months (n, %)	212 (89.8%)	173 (92.5%)
6–12 months (n, %)	23 (9.7%)	14 (7.4%)
Gestational age at birth (n, %)		
<32 weeks	17 (7.2%)	10 (5.3%)
32–37 weeks	26 (11.0%)	37 (19.7%)
>37 weeks	192 (81.4%)	141 (75.0%)
Transport type before admission	227 (96.2%)	174 (92.6%)
MICU (n, %)	110 (46.6%)	84 (44.7%)
Ambulance (n, %)	117 (49.6%)	90 (47.9%)
No transport (n, %)	8 (3.4%)	14 (7.4%)
PICU admission (n, %)		
Length PICU admission (median days, IQR)^b^	5.0 (3.0–8.0)	5.0 (3.0–8.0)
Transport at discharge		
Ambulance (n, %)	178 (75.4%)	138 (73.4%)
None (n, %)	57 (24.3%)	50 (26.6%)
Outpatient clinic visit^c^	98 (41.5%)	71 (37.8%)
With psychological consultation (n, %)	31 (13.1%)	17 (9.0%)
Without psychological consultation (n, %)	67 (28.4%)	54 (28.7%)
No outpatient clinic visit	136 (57.9%)	115 (61.2%)
Readmissions related to initial PICU admission (n, %)	6 (2.5%)	12 (6.4%)
Length PICU readmission (median days, IQR)	4.0 (1.0–9.3)	4.0 (2.0–5.0)

n, number of infants, IQR, interquartile range, PICU, paediatric intensive care unit, MICU, mobile intensive care unit, RSV, respiratory syncytial virus.

a Data of one infant in season one was missing.

b Length of PICU admission for season one had a minimum of one day and a maximum of 28 days. Length of PICU admission for season two has a minimum of one day and maximum of 21 days.

c Data on outpatient clinic visits was missing for 4 (0.9%) infants.

### Baseline characteristics subgroup II and III

In total 98 children had a postponed elective PICU admission (subgroup II). Primary indications for elective PICU admission were post-operative care (62.2%), and other procedures (30.6%). Indications are further specified in Table 2. The number of days between cancellation of the initial elective PICU admission and the newly scheduled PICU admission was available for 25 (25.5%) children. The median number of days the admission postponed was 9 days (IQR 1–28 days).

**Table 2:** Primary indications for PICU admission in children with postponed PICU admission (subgroup II), n (%).

Indication	Children (n = 98)
Surgery	61 (62.2%)
ENT Surgery	16 (16.3%)
Surgery not specified	16 (16.3%)
Cardiac Surgery	9 (9.2%)
Orthopedic Surgery	11 (11.2%)
Plastic and Reconstructive Surgery	5 (5.1%)
GI Surgery	2 (2.0%)
General Surgery	1 (1.0%)
Neurosurgery	1 (1.0%)
Procedure	30 (30.6%)
Polysomnography	12 (12.2%)
Administration/test procedure for special medication	6 (6.1%)
Respiratory monitoring	5 (5.1%)
ICD placement	2 (2.0%)
Bronchoscopy	2 (2.0%)
ICP measurement	1 (1.0%)
Paediatric Oncology Procedure	1 (1.0%)
Setting up chronic ventilation	1 (1.0%)
Antenatal PICU Reservation	7 (7.1%)
Antenal PICU reservation due to known anomalies in the infant during pregnancy	7 (7.1%)

n, number of infants, PICU, paediatric intensive care unit, ENT, Ear, Nose, and Throat, GI, Gastroenterology, ICD, implantable cardioverter-defibrillator, ICP, intracranial pressure.

In total 92 children required an acute transfer to another PICU (subgroup III) of which 58 (63.0%) were MICU transports and 34 (37.0%) were regular ambulance transports. Frequencies per season are shown in Table 3.

**Table 3:** RSV-related healthcare costs on PICUs categorized by season and type of costs.

Characteristics	Season 1 (Sep 2021–Sep 2022)		Season 2 (Oct 2022–June 2023)	
	PICU admitted infants (n = 236)^a^	Total costs	PICU admitted infants (n = 188)	Total costs
Subgroup I^a^	236 (55.7%)	€ 3,756,346.52^b^	188 (44.3%)	€ 3,100,677.65^c^
Costs PICU admission	235 (99.6%)	€ 3,217,558.09	188 (100%)	€ 2,543,954.21
Transport before admission	227 (96.2%)	€ 308,498.30	173 (92.5%)	€ 235,803.00
MICU transport	110 (46.6%)	€ 268,730.00	84 (44.7%)	€ 205,212.00
Ambulance transport	117 (49.6%)	€ 39,768.30	90 (47.9%)	€ 30,591.00
Ambulance at discharge	178 (75.4%)	€ 60,502.20	138 (73.4%)	€ 46,906.20
Costs PICU readmissions	6 (2.6%)	€ 69,443.70	12 (6.4%)	€ 208,331.10
Outpatient clinic visits	98 (41.5%)	€ 85,987.37	70 (37.4%)	€ 65,683.14
Outpatient clinic visits (without NPSA)	31 (13.1%)	€ 14,054.16	17 (9.0%)	€ 7707.12
Outpatient clinic visits (with NPSA)	67 (28.4%)	€ 71,933.21	54 (28.7%)	€ 57,976.02
Subgroup II^d^		NA		NA
Subgroup III^e^	39	€ 70,039.80	53	€ 83,210.80
MICU transports	27 (69.2%)	€ 65,961.00	31 (58.5%)	€ 75,733.00
Ambulance transports	12 (30.8%)	€ 4078.80	22 (41.5%)	€ 7477.80
**Total RSV related healthcare costs**		€ 3,826,386.32		€ 3,183,888.45

PICU, paediatric intensive care unit, MICU, mobile intensive care unit, RSV, respiratory syncytial virus, NPSA, neuropsychological assessment, NA, not available.

a Subgroup I: per patient costs of RSV-related PICU admissions. Data of one infant was imputed with the total median costs of the BRICK study cohort.

b Additional characteristics of subgroup I per patient costs in season 1: mean € 15,916.72, median € 11,573.95, min € 2314.79, max € 64,814.12.

c Additional characteristics of subgroup I per patient costs in season 2: mean € 16,492.97, median € 14,356.85, min € 2654.69, max € 139,468.97.

d Subgroup II: indirect RSV-related postponed elective PICU admissions. No costs available.

e Subgroup III: indirect RSV-related transfer.

### Total RSV-related healthcare costs at PICUs

The total RSV-related healthcare costs were € 7,010,274.77 during the study period. Median per patient costs comprised € 14,356.85 (IQR € 7624.17–€ 21,301.22). Table 3 shows the proportion of costs per season. Costs in season one were mainly attributed to PICU admission days (84.1%), and transfers before admission (8.1%). Most costs in season two were attributed to PICU admission days (79.9%), transfers before admission (7.4%), and readmissions (6.5%). In subgroup III the total transfer costs for children requiring acute PICU admission were € 153,250.60 during the study period. Table 3 shows the proportions of costs per transfer type and per season. Costs of subgroup III attributed 1.8% to the total RSV-related healthcare costs in the first season and 2.6% to the total RSV-related healthcare costs in the second season.

The median per patient costs were similar comparing infants below and above six months of age at PICU admission (p = 0.30). However, median per patient costs were higher in infants below three months of age compared with infants above three months of age at PICU admission (€14,568.54 versus €12,253.75, p = 0.04). Furthermore, infants with comorbidities have higher median per patient costs compared to infants without comorbidities (€15,430.48 versus €14,186.90, p = 0.02). Specifically, infants born premature have higher median per patient costs compared to term born infants (€ 16,501.69 versus € 14,356.85, p = 0.006). Infants with congenital heart disease (CHD), bronchopulmonary dysplasia (BPD) and down syndrome (DS) did not have higher median per patient costs compared to infants without CHD, BPD and DS respectively (p = NS).

### Impact of RSV preventive interventions

The health economic impact of RSV preventive interventions is shown in Table 4. In season one 212/236 (89.9%) infants with RSV-related PICU admissions were below six months of age. In season two 174/188 (92.6%) infants were below six months of age. Potential RSV-related healthcare costs averted by RSV preventive interventions ranges from € 1,976,050.07 to € 2,688,899.33 depending on the season, and the duration of vaccine protection.

**Table 4:** Impact of RSV preventive interventions on direct healthcare costs categorized by season.

Characteristics	Season 1 (Sep 2021–Sep 2022)		Season 2 (Oct 2022–June 2023)	
	PICU admitted infants (n = 236)^a^	Total costs	PICU admitted infants (n = 188)	Total costs
Age at PICU admission ≤3 months (n, %)	175 (74.2%)	€ 2,769,959.33	145 (77.0%)	€ 2,470,062.59
Potential costs averted by RSV preventive intervention^b^		€ 2,215,967.46		€ 1,976,050.07
Age at PICU admission ≤6 months (n, %)	212 (89.8%)	€ 3,361,124.16	174 (92.6%)	€ 2,836,695.86
Potential costs averted by RSV preventive intervention^b^		€ 2,688,899.33		€ 2,269,356.69
Age at PICU admission >6–12 months (n, %)	24 (10.2%)	€ 395,222.36	14 (7.4%)	€ 263,981.79

n, number of infants, PICU, paediatric intensive care unit, RSV, respiratory syncytial virus.

a Data of one infant in season 1 was missing.

b To calculate potential costs averted, we assumed an efficacy of 80% in reducing RSV-related hospitalizations in all infants below three and below six months of age by future RSV preventive interventions (maternal vaccination and infant immunisation) and a vaccine uptake of 100%. For example: total costs of infants ≤3 months of age in season 1 are mutliplied by 80% vaccine efficacy and 100% vaccine uptake.

### Sensitivity analyses

Sensitivity analyses on the impact of RSV preventive interventions showed that minimal potential RSV-related healthcare costs averted ranged from € 1,037,426.29 to € 1,411,672.15 (Table S2).

In addition, in season two one patient was identified with a total per patient cost of € 139,468.97 due to 50 PICU readmission days. Since these costs were exceedingly high compared to all other patients included in the analysis, we performed a sensitivity analysis excluding this patient showing total RSV-related healthcare costs were € 6,936,622.23 during the study period.

## Discussion

The purpose of this multi-center, national, prospective study was to determine the total annual RSV-related healthcare costs at PICUs in the Netherlands. An estimation of the RSV-related health care costs at the PICU is an important step for evaluation of the impact of introduction of RSV preventive interventions into the immunisation programme of the Netherlands. Equally important, this data is imperative for future PICU resource and capacity planning.

Our study shows that the total RSV-related healthcare costs on PICUs is € 3.1–3.8 million in the Netherlands per RSV season. These healthcare costs were primarily related to infants admitted to the PICU with RSV (97%). In 2012 Meijboom et al. estimated that €7.7 million was the total annual societal costs of RSV in the Netherlands of which €3.7 million (48%) was hospital related.^24^ A major difference between these studies in the cost calculation is the total costs used per admission. Meijboom et al. used €3749.36 as the total costs per hospital admission episode, whereas our study used PICU admission costs per day (€ 2314.79), and included all related costs before and after admission. As a result, we found substantially higher total costs per admission with a median of € 14,356.85. In general, unit costs used in the analyses make it challenging to compare total RSV-related healthcare costs across studies. For example, our study used unit costs of € 2314.79 per PICU admission day, while other costs analyses in high income countries used unit costs of €2221–€9174 per ICU admission day^17^^,^^25^ or €3396 per ICU admission episode.^26^

Currently, RSV prevention in the Netherlands is only possible by passive immunisation with Palivizumab.^27^ This anti-F monoclonal antibody is administered monthly only to high risk children and costs €806.87 per unit, and €8.3 million annually in 2022 in the Netherlands.^28^^,^^29^ Due to the high costs Palivizumab is administered solely to infants and children at high risk for severe disease, for example infants born below 32 weeks’ gestation. In our study, we found highest per patient costs in preterm born infants, but only 19 (4.5%) of the PICU admitted infants were born at a gestational age below 32 weeks. This suggests that there is still opportunity to achieve significant reductions in healthcare costs by new, less expensive RSV preventive interventions aiming to prevent RSV disease in all infants.

New RSV preventive interventions could have a substantial impact on the burden of RSV on PICUs because the highest RSV disease burden is observed in children in the first six months of life.^30^ The current study shows that 91% of RSV-related PICU admissions occurred in infants below six months of age. Likewise, the UK showed a median age of two months,^31^ and Australia a median age of four months.^25^ RSV preventive interventions, including maternal vaccination and infant immunisation, target this specific age group. This is important in the light of the rapidly advancing developments in the field.

The health-economic impact of both new interventions has been studied in European cost-effectiveness analyses using different immunisation programmes (e.g. year round, seasonal, catch-up).^17^^,^^18^ Li et al. showed that the annual RSV-related treatment costs in children below five years of age are approximately €8 million in Norway. The cost-effectiveness analysis from the health care payer perspective showed that seasonal mAb programmes were most cost-effective.^18^ Getaneh et al. calculated the potential treatment costs averted by new RSV preventive interventions in six European countries including Scotland, England, Finland, Denmark, Italy and the Netherlands.^17^ The treatment costs averted varied from €665 thousand in Italy to €28.7 million in England depending on the type of intervention, the immunisation programme and the coverage.^17^ The cost-effective analyses from the health care payer perspective showed that the seasonal mAb programme (with or without catch-up) was most cost-effective.^17^ In the Netherlands, the treatment costs averted by new RSV preventive interventions varied from €3.9 to €6.6 million.^17^ In both European studies, treatment costs averted included RSV hospitalisations and primary care cases. To date, no results are available regarding the potential impact of RSV preventive interventions on PICU admissions, and subsequently the costs of PICU admissions. Our study shows that the potential PICU related healthcare costs averted by RSV preventive interventions vary from €1.9 to €2.6 million per season depending on the RSV season and the duration of protection. Future research combining primary care cases, hospitalisations, and PICU admissions is important to understand the full impact of RSV preventive interventions in the Netherlands.

A strength of this study is the prospective real time data collection of all RSV-related PICU admissions and RSV-related transfers, and postponed elective admissions resulting in a precise estimation of total annual health care costs. To elaborate, we were notified weekly by all participating PICUs, and subsequently checked all electronic patient files biweekly for any missing cases. Equally important, we performed a national study. All seven PICUs in the Netherlands participated in the study, because we collaborated with the multicenter national PICU registry (the Dutch paediatric intensive care evaluation, PICE). Furthermore, we have a precise estimation of RSV-attributable PICU admissions as viral testing was routinely performed in all Dutch PICUs after the COVID-19 pandemic.

We are aware that our study may have a number of limitations. First, we did not perform a complete cost-effectiveness analysis as this was beyond the scope of this study. We aimed to determine the total annual healthcare costs of RSV-related PICU admissions as a first step to gain insight in the potential impact of future RSV preventive interventions. To calculate the RSV-related healthcare costs we used unit costs. However, unit costs were not available for outpatient clinic visits. Since DBC costs and unit costs only slightly differed for PICU admission days and transport types, we assumed DBC costs on outpatient clinic visits are an accurate measure of costs. Besides, all costs were extrapolated for the year 2023, to determine the current costs that can potentially be averted assuming an equal rate of all subgroups between 2021 and 2023. In addition, to calculate the potential costs averted we used data of subgroup I only and made assumptions about vaccine efficacy and uptake. The assumed vaccine efficacy is based on previous published trial data that presents the reduction in RSV hospitalisations. We assumed a slightly higher efficacy for more severe outcomes as PICU admission, and a consistent vaccine efficacy over time. In addition, we assumed a vaccine uptake of 100%, although we are aware the vaccine uptake for children is around 90% and for maternal vaccination around 70% in the Netherlands.^32^

Therefore, we performed a sensitivity analysis with assumed lowest vaccine efficacy and lowest vaccine uptake to determine the minimal RSV-related healthcare costs averted. The impact analysis is most probably an underestimation of the potential costs averted, since consequential costs of subgroup II and III were not included in the impact analyses. Second, our study was developed with the healthcare perspective under the assumption that indirect costs including parent loss of productivity and parental transfer would account for a limited amount of money. In the Netherlands we have a 3-months maternity leave and a 6-week paternity leave. Given that we observed a median age of 1.5 months in infants with RSV-related PICU admission, it is conceivable that there was very limited productivity loss by caregivers due to RSV illness in their infants. Third, admissions to general paediatric wards involving outreach from PICU resources and re-admissions to general paediatric wards after PICU discharge were left out of this study due to difficulties in data collection outside the participating PICUs. Admissions with PICU outreach and readmissions to general wards could have increased the total amount of RSV-related healthcare costs. In addition, we did not include in-hospital transfers (e.g. in case a patient is transferred from the general ward to the PICU within the same hospital) since there are no additional costs related to in-hospital transfers. Fourth, we notice some uncertainty in data on postponed elective PICU admissions and acute transfers. Postponed elective PICU admissions and acute transfers were registered as attributable to RSV when one infant with RSV was admitted to the PICU at the same day. There is a possibility that there were other reasons for postponing elective admissions or transferring patients in need for acute PICU admissions, for example lack of personnel. Despite the limitation of this methods, we can still state that preventing one infant from RSV-related PICU admission, will save a PICU bed for another child. In addition, postponed elective admissions are assumed to result in increased healthcare costs. The median number of days between the cancelled admission and the newly scheduled admission was approximately 1.5 week. Throughout this period, disease severity could increase. Also, postponed elective surgeries could lead to increased healthcare costs due to loss of surgical theatre capacity in case the cancelled surgery cannot be replaced due to last-minute cancellation. Unfortunately, no data is available on costs of postponed elective admissions. Therefore, the consequential costs of infants with RSV-related PICU admission could be underestimated. To calculate the potential RSV-related healthcare costs averted we used costs of subgroup I only. Fifth, purchasing and administration costs of new preventive interventions were not accounted for in this study since those costs are not included in the hospital perspective. Future studies performing a complete cost-effectiveness analysis are important to determine potential costs averted from different perspectives. Sixth, we aimed to gain insight in the potential impact of future RSV preventive interventions in the Netherlands. Due to differences between countries, results may not be generalizable. However, results of the current study may provide insights for other high income countries with similar healthcare facilities and structure. Lastly, this study was performed during the COVID-19 pandemic which has changed the “normal” RSV seasonality. This could have impacted the number of RSV-related PICU admissions and consequential transfers and postponed elective admissions. We demonstrated a rate of 188–236 RSV-related PICU admissions during one season. This number of PICU admissions is comparable with PICU admission rates between 2012 and 2016 in infants ≤24 months of age in the Netherlands.^33^ Although, we expected to have less RSV-related PICU admissions because we collected data on infants ≤12 months of age to determine of the health-economic impact of preventive strategies in this age category, but the COVID-19 pandemic resulted in a continuation of RSV epidemic throughout the year in the Netherlands. However, the study of Linssen et al. showed an increased number of RSV-related PICU admissions between 2003 and 2016. This increase was primarily due to increased admissions in children up to three months of age, which is still the targeted age group of RSV preventive interventions.^33^

This study is the first to determine RSV-related healthcare costs of the PICUs in the Netherlands. In summary and under the current assumptions, the introduction of two RSV preventive interventions, maternal vaccination and infant immunisation into the Dutch immunisation programme will generate significant cost-savings on PICUs. Moreover, as the prevention of RSV infection may decrease general RSV-related hospital admission rates, this will most likely decrease the pressure on healthcare resources on an even larger scale. Future clinical data of RSV burden after implementation of RSV preventive interventions will show the accuracy of the current analysis and conclusion.

## Contributors

EP, JGW, MH, LB, JvW, GF contributed to the study's conceptualization and EP, JGW, LB, GF contributed to the study design. The entire team contributed to data collection, and EP, JGW and GF performed the statistical analyses. The first drafts of the manuscript were written by EP, JGW and GF and all authors reviewed and edited subsequent versions of the manuscript. All authors have read and approved the final version of the manuscript. The entire study was supervised by JGW, LB and GF.

## Data sharing statement

Study data including individual participant data that underlie the results reported in this article (after de-identification), the study protocol and the analytic code will be available immediately following publication, no end date. Requests to access the data should be directed to j.g.wildenbeest@umcutrecht.nl; to gain access, data requestors will have to provide a methodologically sound proposal and need to sign a data access agreement.

## Declaration of interests

EP, CB, MH, MK, SM, AN, MR, RWA, JvW and GF have no competing interests to declare.

LB has regular interaction with pharmaceutical and other industrial partners. He has not received personal fees or other personal benefits. UMCU has received major funding (>€100,000 per industrial partner) for investigator initiated studies from AstraZeneca, Sanofi, Janssen, Pfizer, MSD and MeMed Diagnostics. UMCU has received major funding from the Bill and Melinda Gates Foundation. UMCU has received major funding as part of the public private partnership IMI-funded RESCEU and PROMISE projects with partners GSK, Novavax, Janssen, AstraZeneca, Pfizer and Sanofi. UMCU has received major funding by Julius Clinical for participating in clinical studies sponsored by AstraZeneca, Merck and Pfizer. UMCU received minor funding (€1000–25,000 per industrial partner) for consultation, DSMB membership or invited lectures by Ablynx, Bavaria Nordic, GSK, Novavax, Pfizer, Moderna, Astrazeneca, MSD, Sanofi, Janssen. LB is the founding chairman of the ReSViNET Foundation.

JGW has been an investigator for clinical trials sponsored by pharmaceutical companies including AstraZeneca, Merck, Pfizer, Sanofi, and Janssen and an investigator for clinical trials funded by IMI/Horizon2020 and ZonMw. All funds have been paid to the institution (UMCU). JGW participated in advisory boards of Janssen and Sanofi and was a speaker at a Sanofi sponsored symposium with fees paid to the institution (UMCU).
