# Supplementary material for: Healthcare costs related to respiratory syncytial virus in paediatric intensive care units in the Netherlands: a nationwide prospective observational study (the BRICK study)
Source: Lancet Reg Health Eur. 2024 Jun 26;43:100965. doi: 10.1016/j.lanepe.2024.100965 (PMC11260872; doi:10.1016/j.lanepe.2024.100965)
Supplement: Supplementary Tables [file mmc1.docx]

**Supplementary material**

Healthcare costs related to respiratory syncytial virus in paediatric intensive care units in the Netherlands: a nationwide prospective observational study (the BRICK study)

**Content**

| Table S1 | Costs per unit for the year 2023, expressed in €. | Page 2 |
| --- | --- | --- |
| Table S2 | Minimal impact of RSV preventive interventions on direct healthcare costs categorized by season. | Page 3 |

| **Table S1: Costs per unit for the year 2023, expressed in €.** | |
| --- | --- |
| **Unit** | **Unit costs*** |
| PICU admission day | € 2,314·79 |
| MICU transport | € 2,443·00 |
| Ambulance transport | € 339·90 |
| Outpatient clinic visit | € 453·26 |
| Outpatient clinic visit (with NPSA) | € 1,073·63 |
| PICU = paediatric intensive care unit, MICU = mobile intensive care unit, NPSA=neuropsychological assessment.  * Unit costs are all costs associated with specific activities and/or treatment for individual patients (costs from 2023). In case unit costs were missing or unknown we used DBC costs. DBC costs are the maximum costs a healthcare provider can charge for specific patient groups according to the Dutch Health Care Insitute (ZIN) costing manual (Hakkaart-van Roijen L, Van der Linden N, Bouwmans CAM, Kanters TA, 2015; Kanters et al., 2017) (costs from 2023). | |

| **Table S2: Minimal impact of RSV preventive interventions on direct healthcare costs categorized by season** | | | | |
| --- | --- | --- | --- | --- |
|  | **Season 1 (Sep 2021 – Sep 2022)** | | **Season 2 (Oct 2022 – June 2023)** | |
| **Characteristics** | **PICU admitted infants (n=236)*** | **Total costs** | **PICU admitted infants (n=188)** | **Total costs** |
| Age at PICU admission ≤3 months (n, %) | 175 (74·2%) | € 2,769,959·33 | 145 (77·0%) | € 2,470,062·59 |
| Potential costs averted by RSV preventive intervention ˟ |  | € 1,163,382·92 |  | € 1,037,426·29 |
|  |  |  |  |  |
|  |  |  |  |  |
|  |  |  |  |  |
| Age at PICU admission ≤6 months (n, %) | 212 (89·8%) | € 3,361,124·16 | 174 (92·6%) | € 2,836,695·86 |
| Potential costs averted by RSV preventive intervention ˟ |  | € 1,411,672·15 |  | € 1,191,412·26 |
|  |  |  |  |  |
|  |  |  |  |  |
|  |  |  |  |  |
| Age at PICU admission >6-12 months (n, %) | 24 (10·2%) | € 395,222·36 | 14 (7·4%) | € 263,981·79 |
| n = number of infants, PICU = paediatric intensive care unit, RSV = respiratory syncytial virus. * Data of one infant in season 1 was missing. ˟ To calculate potential costs averted, we assumed an efficacy of 60% in reducing RSV-related hospitalizations in all infants below three and below six months of age by future RSV preventive interventions (maternal vaccination and infant immunisation) and a vaccine uptake of 70%. For example: total costs of infants ≤3 months of age in season 1 are mutliplied by 60% vaccine efficacy and 70% vaccine uptake. | | | | |
